# Recurrent Ventricular Fibrillation Associated With Graves′ Disease: A Case Report

**DOI:** 10.1155/cric/6567826

**Published:** 2026-06-28

**Authors:** In Tae Moon, Sihun Kim, Sung Hun Park, You-Jeong Ki, Jung Yeon Chin

**Affiliations:** ^1^ Division of Cardiology, Department of Internal Medicine, Uijeongbu Eulji University Hospital, Uijeongbu-si, Gyeonggi-do, Republic of Korea

**Keywords:** case report, Graves′ disease, implantable cardioverter defibrillator, radioactive iodine-131, thyrotoxicosis, ventricular fibrillation

## Abstract

Although tachyarrhythmias are associated with thyrotoxicosis, the association with ventricular fibrillation is unknown. This case reports a recurrent ventricular fibrillation with Graves′ disease that was successfully treated with an implantable cardioverter defibrillator. A 33‐year‐old woman presented with ventricular fibrillation cardiac arrest. She was diagnosed with Graves′ disease and was taking antithyroid drugs, but her hyperthyroidism was poorly controlled. The patient received intensive care unit care, including extracorporeal membrane oxygenator, ventilator, and renal replacement therapy. Implantable cardioverter defibrillator was implanted for the secondary prevention of ventricular fibrillation. Two months after the procedure, ventricular fibrillation recurred, and an implantable cardioverter defibrillator successfully prevented sudden cardiac death. She maintained a normal sinus rhythm and normal thyroid function after receiving radioactive iodine‐131 therapy. Ventricular fibrillation is a rare but fatal manifestation in patients with hyperthyroidism. Implantable cardioverter defibrillator should be considered, especially in patients with poorly controlled hyperthyroidism, for the prevention of sudden cardiac death.

## 1. Introduction

Thyroid hormone increases protein synthesis, resulting in ventricular hypertrophy; dilates vessels, resulting in tachycardia and widened pulse pressure; and increases sympathetic activity [1]. Thyrotoxicosis may contribute to the development of sinus tachycardia or atrial tachyarrhythmia [2]. Five percent to 15% of patients with hyperthyroidism have atrial fibrillation, which is associated with cardiovascular complications [3].

However, its association with ventricular fibrillation (VF) is uncertain [4]. Ventricular arrhythmias may also be associated with hypothyroidism [5], and the prevalence in thyrotoxic subjects remains unchanged during and after antithyroid therapy [6]. Although there are a few case reports of thyrotoxicosis‐induced cardiac arrest [7, 8], including VF, recurrence of ventricular arrhythmia has not been reported. The majority of previous cases of VF induced by thyrotoxicosis occur in the context of thyrotoxic periodic paralysis with severe hypokalemia [9]; a case of early repolarization‐associated VF has also been reported [10]. But the authors have not concluded the mechanisms of VF in hyperthyroidism.

Here, we report a case of recurrent VF without a predisposing condition that was successfully prevented from sudden cardiac death using an implantable cardioverter defibrillator (ICD).

## 2. Case Presentation

A 33‐year‐old woman presented to the emergency department with a cardiac arrest. She was diagnosed with Graves′ disease 8 years prior to presentation and was treated with methimazole (40 mg/day) for 18 months. Graves′ disease was confirmed with a thyroid‐stimulating hormone (TSH) receptor antibody level greater than 40 IU/L and a thyroid scan showing diffuse increased uptake. Her baseline electrocardiography (ECG) 15 months prior to the event showed a normal sinus rhythm, and baseline echocardiography showed normal left ventricular ejection fraction (LVEF). Atrial fibrillation with a rapid ventricular response developed 2 months prior to presentation. Treatment with furosemide 80 mg, spironolactone 25 mg, and bisoprolol 10 mg per day was initiated to control atrial fibrillation and generalized edema. Considering her CHADS2‐VASc score was 2 points, with 1 point attributed to congestive heart failure and 1 point attributed to sex, an anticoagulant was not prescribed. Her free thyroxine (free T4) level was 7.21 ng/dL (reference range: 0.89~1.76) at that time, which had decreased from the previous measurement (11.8 ng/dL), but it was still significantly high. Follow‐up echocardiography was planned after her heart rate was controlled. External electrical cardioversion was not considered because the main cause of atrial fibrillation was uncontrolled hyperthyroidism.

**Figure 1:**
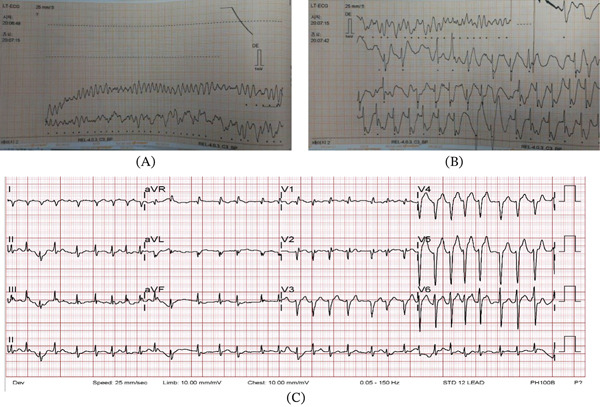
(A) Initial electrocardiography (ECG) rhythm on the automated external defibrillator showed ventricular fibrillation and a shockable rhythm. (B) After defibrillation, the ECG rhythm returned to atrial fibrillation. (C) Initial 12‐lead ECG at the emergency department. ECG shows atrial fibrillation with rapid ventricular response, with a ventricular rate of 192.

The initial VF detected at the emergency rescue service returned to atrial fibrillation after 200 J defibrillation was performed (Figure 1). Her body temperature was 36.7°C, heart rate was 192 bpm (beats per minute), respiration rate was 30 breaths/min, and blood pressure was 41/30 mmHg. ECG performed at the hospital revealed atrial fibrillation with a heart rate of 192 bpm. She had a Glasgow Coma Scale score of E1V1M1 (no eye opening, verbal response, and motor response), and neurological examination showed no pupil response or brainstem reflexes.

Since she had no underlying cardiovascular disease, VF associated with hyperthyroidism was strongly suspected. Coronary vasospasm or torsade de points due to hypokalemia had to be ruled out.

**Figure 2:**
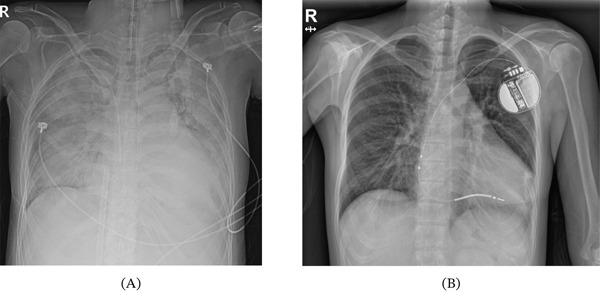
(A) Chest X‐ray on the day of admission revealed cardiomegaly and severe pulmonary edema. The patient was intubated, and an extracorporeal membrane oxygenator and central lines were inserted. (B) Chest X‐ray after insertion of an implantable cardioverter defibrillator revealed resolved pulmonary edema.

Laboratory findings and the reference ranges are summarized in Table 1. TSH was below the detectable limit, and free T4 was markedly elevated. Her electrolyte levels were within the normal range, and there were no significant changes before or after the cardiac arrest. Chest radiography revealed severe pulmonary edema and marked cardiomegaly (Figure 2A). Bedside echocardiography showed severely decreased biventricular systolic function. LVEF was about 10% with global severe hypokinesia (Supporting Information, Video S1).

**Table 1:** Laboratory findings and the reference ranges at the time of cardiac arrest.

Variables	Value	Reference range
White blood cell count (count/*μ*L)	10,140▲	4000~10,000
Hemoglobin (g/dL)	12.1	12.0~16.0
Platelet (count/*μ*L)	173,000	130,000~450,000
Sodium (mmol/L)	135▼	136~145
Potassium (mmol/L)	4.7	3.5~5.1
Chloride (mmol/L)	100	98~107
Calcium (mg/dL)	8.6	8.3~10.6
Phosphorus (mg/dL)	7.7▲	2.4~5.1
Blood urea nitrogen (mg/dL)	8.7▼	9~23.0
Creatinine (mg/dL)	0.8	0.55~1.02
Estimated GFR (mL/min/1.73m^2^)	83	
AST (U/L)	34	0~34
ALT (U/L)	11	10~49
pH	7.164▼	7.320~7.450
PaCO_2_ (mmHg)	51.9▲	32.0~48.0
PaO_2_ (mmHg)	82.8	83.0~108.0
HCO_3_ (mEq/L)	18.3	18~23
Lactate (mmol/L)	7.08▲	0.36~1.39
*N*‐terminal pro‐BNP (pg/mL)	2804▲	
Troponin‐I (ng/mL)	0.01	0~0.0341
C‐reactive protein	< 0.300	0~0.3

Thyroid function tests		
Thyroid‐stimulating hormone (TSH) (uIU/mL)	< 0.01▼	0.55~4.78
Free thyroxine (free T4) (ng/dL)	6.81▲	0.89~1.76
Triiodothyronine (T3) (ng/dL)	411.4▲	60~181
TSH receptor antibody (IU/L)	22.1▲	0~1.75
Thyroid‐stimulating antibody (%)	360.0▲	< 140
Thyroid microsomal antibody (U/mL)	597.5▲	0~60

*Note:* ▲, upper than normal reference range; ▼, lower than normal reference range.

Abbreviations: ALT, alanine aminotransferase; AST, aspartate aminotransferase; BNP, brain natriuretic peptide; GFR, glomerular filtration rate; HCO_3_, bicarbonate; PaCO_2_, partial pressure of arterial carbon dioxide; PaO_2_, partial pressure of arterial oxygen; pH, percentage of hydrogen ions; TSH, thyroid‐stimulating hormone.

She received full postreturn spontaneous circulation care, including mechanical ventilation, continuous renal replacement therapy, extracorporeal membrane oxygenation, and therapeutic hypothermia. The extracorporeal membrane oxygenator was successfully removed on the fifth day of admission, and she was extubated the following day. Her ECG rhythm maintained atrial fibrillation but a tolerable heart rate with bisoprolol 7.5 mg/day. The endocrinologist recommended increasing the dose of methimazole from 40 to 60 mg/day, and iodine solution therapy was started.

Coronary artery disease was not detected on the coronary angiography with an ergonovine provocation test. Echocardiography showed improved LVEF from 10% to 48% at Hospital Day 12 (Supporting Information, Video S2). Based on these findings, poor R‐wave progression and right‐axis deviation of the ECG are considered nonspecific. We failed to obtain a cardiac magnetic resonance imaging due to an irregular heartbeat and a poorly controlled heart rate below 70 bpm despite the maximal dose of a beta blocker. Cardiomyopathy or myocarditis was excluded based on echocardiographic findings and clinical features. Genetic testing for cardiac arrhythmias, including SCN5A and long QT syndrome, was all negative. She had no history of syncope, nor a family history of sudden cardiac death, cardiomyopathy, or channelopathy.

Transvenous ICD was implanted on the 14th day for the secondary prevention of VF (Figure 2B). The patient was discharged with an atrial fibrillation rhythm with a heart rate of 80 bpm. Her free T4 level was 1.97 ng/dL (reference range: 0.89~1.76). Her clinical course and corresponding thyroid function tests, along with the timeline, are summarized in Table 2.

**Table 2:** Timeline of clinical course and thyroid function tests.

Timeline	Clinical course	Thyroid function test
		(TSH/free T4)
15 months	First visited our center with palpitations.	0.006/9.49
	She had been diagnosed with hyperthyroidism 7 years earlier but had not received treatment for 1 year. ECG showed sinus tachycardia with a heart rate of 115 bpm, and echocardiography revealed a normal left ventricular ejection fraction and a left atrial volume index of 70 mL/m^2^	
2 months	Lost to follow‐up for 13 months	0.006/11.18
	Atrial fibrillation with rapid ventricular response was first detected. She complained of dyspnea, generalized edema, and worsening palpitations	

Day 0	Ventricular fibrillation with cardiac arrest	< 0.008/6.81
Day 4	Extracorporeal membrane oxygenator removed	< 0.008/6.81
Day 6	Extubated	< 0.008/4.83
Day 14	Implantable cardioverter defibrillator inserted	< 0.008/2.87
Day 40	Discharged	< 0.008/1.97
Day 55	Ventricular fibrillation recurred	Not checked
Day 62	Received radioactive iodine‐131 therapy	< 0.008/7.61
4 months	Sinus rhythm restored, left ventricular function normalized	< 0.008/1.16

*Note:* Reference range: TSH (0.55~4.78 uIU/mL), free T4 (0.89~1.76 ng/dL).

Abbreviations: ECG, electrocardiography; free T4, free thyroxine; TSH, thyroid‐stimulating hormone.

**Figure 3:**
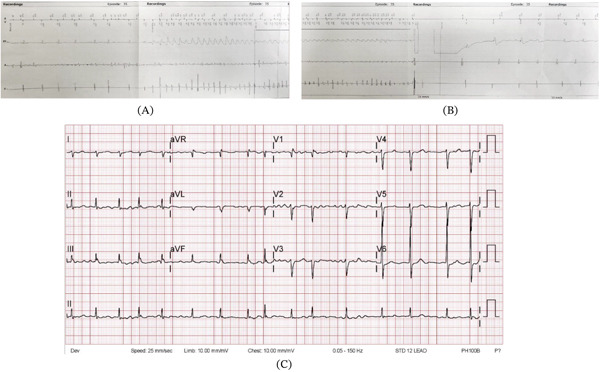
(A) Recurrent ventricular fibrillation episode recorded at an implantable cardioverter defibrillator (ICD). Ventricular fibrillation occurred 2 months after ICD implantation. (B) ICD successfully performed defibrillation with 30 J energy. (C) A 12‐lead electrocardiography immediately after defibrillation. It shows atrial fibrillation rhythm, and there is no evidence of ST‐T wave change or QTc prolongation.

Two months after ICD implantation, VF recurred, and the ICD successfully performed defibrillation (Figure 3A,B). Her ECG rhythm after defibrillation was atrial fibrillation (Figure 3C). The free T4 level checked 6 days before the recurrence of VF was 1.66 ng/dL (reference range: 0.89~1.76), and 14 days afterward, the free T4 level was 7.61 ng/dL. Although the exact value of free T4 level at the time of VF could not be known, it might have been above the normal range. She was still taking methimazole at a dose of 60 mg/day and did not skip any drugs.

After her general condition recovered, she received radioactive iodine‐131 (RAI) therapy at a dose of 15 mCi, and her thyroid function was normalized with a maintenance dose of methimazole (15 mg/day). Her ECG maintained a normal sinus rhythm, and echocardiography showed normal LVEF after normalization of thyroid function. She had no recurrent episodes of VF with bisoprolol 2.5 mg/day for 2 years.

## 3. Discussion

This is a case of VF associated with hyperthyroidism without structural heart disease, even after the treatment for Graves′ disease. The patient′s hyperthyroidism was not well controlled despite antithyroid drug use, atrial fibrillation developed thereafter, and cardiac arrest developed due to VF.

Excess thyroid hormones can cause sinus tachycardia and atrial fibrillation, as well as increase ventricular contractile function and peripheral oxygen consumption [3]. The mechanism of atrial fibrillation involves shortening of the action potential duration in the atrial myocardium and a decrease in the refractoriness of cardiomyocytes [11]. In contrast, VF is a rare manifestation of hyperthyroidism, and its underlying mechanism is unclear.

Although thyroid hormones do not directly induce VF, there are a few known mechanisms by which hyperthyroidism causes ventricular arrhythmia. First, VF can be caused by coronary vasospasm [12], and severe coronary vasospasm could be associated with hyperthyroidism [13]. Second, significant hypokalemia with thyrotoxic period paralysis can cause life‐threatening ventricular arrhythmias [14]. Third, patients with hyperthyroidism may experience symptoms of heart failure and myocardial dysfunction [3]. Although hyperthyroid cardiomyopathy has not been shown to cause ventricular arrhythmia, heart failure with a reduced LVEF generally increases the risk of sudden cardiac death and ventricular arrhythmia [15].

The patient had normal potassium levels, relatively preserved LVEF, and no evidence of coronary vasospasm. Some cases of VF have been reported in thyrotoxicosis in the absence of hypokalemia, heart failure, or underlying cardiovascular disease [9], whereas recurrent VF cases have not been reported. The role of the ICD in previous cases was questionable because the recurrence rate of VF in patients with hyperthyroidism is unclear. However, a prospective population‐based study suggested that high free T4 levels are associated with sudden cardiac death, and the need for ICD implantation is warranted for secondary prevention [16].

There are several issues related to the differential diagnosis in this case. First, tachycardia‐induced cardiomyopathy and other kinds of nondilated cardiomyopathy were excluded based on the clinical course. Although the patient complained of generalized edema 2 months before the occurrence of VF, she had tolerable symptoms while taking diuretics and did not experience any worsening of symptoms up until just before cardiac arrest. Her LVEF rapidly improved from 10% to 48% on the 12th hospital day. Four months after the occurrence of VF, a follow‐up echocardiography showed that heart function, including LVEF, was within normal range, and a normal sinus rhythm was maintained. If severe left ventricular dysfunction due to tachycardia had preceded, she would have experienced worsening dyspnea before visiting the emergency department. If other kinds of cardiomyopathy had coexisted, her left ventricular function would not have normalized without definitive treatment. Since her cardiac function fully recovered with thyroid treatment alone, without any specific therapy for cardiomyopathy, hyperthyroidism is considered the possible cause of her VF. Second, there is a possibility of an occult accessory pathway existing. However, upon reviewing all her ECG records, no delta waves suggestive of manifested accessory pathway were observed, and she had no symptoms or clinical events suggestive of atrioventricular reentry tachycardia.

Patients with Graves′ disease should be treated with either RAI therapy, antithyroid drugs, or thyroidectomy [4]. Definitive treatment, including RAI therapy and thyroidectomy, is preferred when antithyroid drugs are ineffective or when the thyroid storm requires rapid treatment. However, as the patient reported here was relatively young and had the symptoms of heart failure and atrial fibrillation, the endocrinologist did not decide on early thyroidectomy, considering her age and surgical risk. The patient had a normal free T4 level, and sinus rhythm was maintained after RAI therapy followed by methimazole. If RAI therapy had been administered earlier, cardiac arrest due to VF could have been avoided at the time of uncontrolled hyperthyroidism despite the highest dose of antithyroid drugs.

Then, there may be debate about the necessity of implanting an ICD for VF caused by reversible hyperthyroidism. Since the patient had a poor response to high‐dose antithyroid medication, the effectiveness of RAI therapy could not be guaranteed. In this situation, the risk of VF recurrence persisted. Because thyroid storm may also cause ventricular tachycardia [17], we implanted a transvenous ICD capable of antitachycardia pacing to terminate ventricular tachycardias.

Since QT prolongation is a marker of potential ventricular arrhythmia and sudden cardiac death, frequent QT interval monitoring has been suggested as an alternative approach [18]. Self‐monitoring of the QT interval may be beneficial in some patients; however, her baseline QTc interval was 457 ms, and considering that she had an atrial fibrillation rhythm, its usefulness is questionable.

VF with hyperthyroidism is a rare clinical manifestation but is clinically important and potentially life‐threatening. ICD implantation should be considered to prevent sudden cardiac death in patients with uncontrolled hyperthyroidism who experience VF. Even if a patient fully recovers from cardiac arrest, secondary prevention is necessary, as uncontrolled thyroid function carries a risk of recurrent VF. Aggressive antithyroid therapy, including RAI therapy and thyroidectomy, is also required to improve clinical outcomes.

**Nomenclature**
BPM: beats per minute
ECG: electrocardiography
ICD: implantable cardioverter defibrillator
LVEF: left ventricular ejection fraction
RAI: radioactive iodine‐131
TSH: thyroid‐stimulating hormone
Free T4: free thyroxine
VF: ventricular fibrillation

## Author Contributions

In Tae Moon: writing (original draft, review, and editing), conceptualization, data curation, methodology, supervision, and funding acquisition. Sihun Kim: writing (original draft), investigation, software, and methodology. Sung Hun Park: writing (original draft), formal analysis, and project administration. You‐Jeong Ki: writing (review and editing), project administration, and validation. Jung Yeon Chin: writing (review and editing), resources, and visualization.

## Funding

No funding was received for this manuscript.

## Disclosure

The authors presented this case report at “The 67th Annual Scientific Meeting of the Korean Society of Cardiology.”

## Consent

Written informed consent has been obtained from the patient to publish this paper.

## Conflicts of Interest

The authors declare no conflicts of interest.

## Supporting Information

Additional supporting information can be found online in the Supporting Information section.

## Supporting information


**Supporting Information 1** Video S1: Bedside echocardiography performed just after the extracorporeal membrane oxygenator was inserted. It showed severely decreased systolic function with a left ventricular ejection fraction of about 10%.

**Supporting Information 2** Video S2: Echocardiography performed at Hospital Day 12. It showed markedly improved systolic function with a left ventricular ejection fraction measured 48%.

## Data Availability

All data generated or analyzed during this study are included in this article. Further enquiries can be directed to the corresponding author.
